# Supplementary material for: In Silico and In Vitro Analyses Validate Human MicroRNAs Targeting the SARS-CoV-2 3′-UTR
Source: Int J Mol Sci. 2021 Jun 5;22(11):6094. doi: 10.3390/ijms22116094 (PMC8201247; doi:10.3390/ijms22116094)
Supplement: Supplementary file 1 [file ijms-22-06094-s001.zip › Legends of Supplementary Tables.pdf]

Table S1. Human microRNAs (miRbase release 22) targeting SARS-CoV-2 3′-UTR. Excel file containing the predicted binding sites of human microRNAs in the SARS-CoV-2 3′-UTR according to miRDB, IntaRNA, RNA22, RNAhybrid and STarMir prediction tools. Input data were the reference sequence of SARS-CoV-2 from Wuhan (China, GenBank accession NC_045512.2) and all human microRNAs from the miRbase release 22. The first 5 sheets in the file include the predictions and associated data obtained from each tool. To reduce computational load, only those microRNA identified by miRDB prediction tools were analyzed with the other tools. The last sheet (List of all microRNAs) summarizes the list of microRNAs predicted by all tools (except for RNA22 due to its strong inconsistencies with the other predictors). Nucleotide positions are referred to the 3′-UTR without the poly-A tail (i.e. nt 1 to 196) except for STarMir which required the last nucleotide from the coding region for analysis and, therefore, the 3′-UTR positions correspond to nt 2 to 197.

Table S2. High confidence human microRNAs (miRbase release 21) targeting SARS-CoV-2 3′-UTR. Excel file containing the predicted binding sites of human microRNAs in the SARS-CoV-2 3′-UTR according to IntaRNA, RNA22, RNAhybrid and STarMir prediction tools. Input data were the reference sequence of SARS-CoV-2 from Wuhan (China, GenBank accession NC_045512.2) and the human microRNAs classified as high confidence according to miRbase release 21 (see details in http://www.mirbase.org/blog/2014/07/high-confidence-mirna-set-available-for-mirbase-21/). The first 4 sheets in the file include the predictions and associated data obtained from each tool. There is no miRDB sheet because it did not provide predictions for any high-confidence human microRNA. The last sheet (List of high confidence) summarizes the list of microRNAs predicted by all tools (except for RNA22 due to its strong inconsistencies with the other predictors). Nucleotide positions are referred to the 3′-UTR without the poly-A tail (i.e. nt 1 to 196) except for STarMir which required the last nucleotide from the coding region for analysis and, therefore, the 3′-UTR positions correspond to nt 2 to 197.

Table S3. Sequences from SARS-CoV-2 variants and related virus employed in the sequence comparisons. 60 sequences comprising all clades recognized in GISAID, sampled in 32 countries from November 2019 to February 2021. Only sequences comprising the 196 nt of the 3′-UTR have been considered. Clades correspond to those recognized in GISAID. Variant indicates the GISAID Pangolin Lineages (PL) or the variant of each sequence (* indicates the South African variant, ** the British variant, *** the Brazilian variant, and **** the Californian variant). ID indicates accession ID of GenBank (first 6 sequences) or GISAID (EPI_ …). Collect date indicates the date of collection. Identity (%) expresses the % of sequence identity relative to the reference sequence (NC_045512.2) according to CLUSTALW online tool (https://www.genome.jp/tools-bin/clustalw). Reference includes the article where the sequence is published or, when unpublished, information on the responsible for the collection, analysis, and submission of the sequences. No data on the sequence is published in agreement with the terms and conditions of use of GISAID.
