# Supplementary material for: In Silico and In Vitro Analyses Validate Human MicroRNAs Targeting the SARS-CoV-2 3′-UTR
Source: Int J Mol Sci. 2021 Jun 5;22(11):6094. doi: 10.3390/ijms22116094 (PMC8201247; doi:10.3390/ijms22116094)
Supplement: Supplementary file 1 [file ijms-22-06094-s001.zip › Table S3.pdf]

**Table S3.** Sequences from SARS-CoV-2 variants and related virus employed in the sequence comparisons. 60 sequences comprising all clades recognized in GISAID, sampled in 32 countries from November 2019 to February 2021. Only sequences comprising the 196 nt of the 3′-UTR have been considered. Clades correspond to those recognized in GISAID. Variant indicates the GISAID Pangolin Lineages (PL) or the variant of each sequence (* indicates the South African variant, ** the British variant, *** the Brazilian variant, and **** the Californian variant). ID indicates accession ID of GenBank (first 6 sequences) or GISAID (EPI_ …). Collect date indicates the date of collection. Identity (%) expresses the % of sequence identity relative to the reference sequence (NC_045512.2) according to CLUSTALW online tool (https://www.genome.jp/tools-bin/clustalw). Reference includes the article where the sequence is published or, when unpublished, information on the responsible for the collection, analysis, and submission of the sequences. No data on the sequence is published in agreement with the terms and conditions of use of GISAID.

| Clade | Variant | ID | Origin | Collect Date | Identity (%) | Reference |
|---|---|---|---|---|---|---|
| | Reference Genome | NC_045512.2 | Wuhan (China) | 2019-11 | REF | Wu *et al.*, 2020 |
| | Original | LC546038.1 | Japan | 2020-03 | 100% | https://www.ncbi.nlm.nih.gov/nuccore/LC546038.1 |
| | D614G mutation | MT192773.1 | Ho Chi Minh (Vietnam) | 2020-01 | 100% | https://www.ncbi.nlm.nih.gov/nuccore/MT192773.1 |
| | Original | MT192759.1 | Taiwan | 2020-01 | 100% | https://www.ncbi.nlm.nih.gov/nuccore/MT192759 |
| | Unknown | MT114415 | Hong Kong (China) | 2020-01 | 99% nt 29742 G->U | The University of Hong Kong \| To KKW and Yuen K-Y |
| | Unknown | MT370518.1 | Taiwan | 2020-02 | 99% nt 29742 G->U | Department of Laboratory Medicine, Lin-Kou Chang-Gung Memorial Hospital \| K-C Tsao, Y-N Gong *et al.* |
| GH | PL B.1.2 | EPI_ISL_812206 | Tennessee (USA) | 2020-11 | 100% | TN Division of Laboratory Services \| Centers for Disease Control and Prevention \| Y Li, Y Tao *et al.* |
| GH | PL B.1.247 | EPI_ISL_490001 | Saudi Arabia | 2020-02 | 100% | King Fahad Medical City \| Alosaimi,B., Naeem,A., Alghoraibi,M., Enani,M. |
| GH | PL B.1.36.18 | EPI_ISL_853274 | Toronto (Canada) | 2020-12 | 100% | Microbiology Lab, Mount Sinai Hospital \| NBCC Sequencing Facility \| J Wrana. |
| GH | PL B.1 | EPI_ISL_802774 | Galicia (Spain) | 2020-10 | 100% | Hospital Santa María Nai \| Instituto de Salud Carlos III \| M Iglesias-Caballero, M Molinero-Calamita *et al.* |
| GH | PL B.1.36 | EPI_ISL_859730 | Abu Dhabi (UAE) | 2020-09 | 100% | *B*TC, Khalifa University \| Al Safar *et al* |
| GH | PL B.1.351* | EPI_ISL_700461 | George (South Africa) | 2020-11 | 95% gap nt 29862-29869 | Thembalethu CDC wc THC \| NHLS/UCT \| Tegally H, Iranzadeh A *et al* . |

| | | | | | | |
|---|---|---|---|---|---|---|
| GH | PL B.1.351* | EPI_ISL_860577 | Zululand (South Africa) | 2021-01 | 94% gap nt 29862-29869 | NHLS-IALCH ǀ KRISP, KZn Research Innovation and Sequencing Platform ǀ Giandhari J, Pillay S *et al.* |
| GH | PL B.1.351* | EPI_ISL_736980 | Sarah Baartman (South Africa) | 2020-12 | 96% gap nt 29864-29870 | NHLS-IALCH ǀ KRISP, KZn Research Innovation and Sequencing Platform ǀ Giandhari J, Pillay S *et al.* |
| GH | B.1.427**** | EPI_ISL_824620 | California (USA) | 2020-12 | 100% | Cedars-Sinai Medical Center, Molecular Pathology Laboratory of Department of Pathology & Laboratory Medicine and Genomic Core ǀ Zhng, Davis *et al* |
| GH | B.1.429 + B.1.427**** | EPI_ISL_960573 | Campania (Italy) | 2021-01 | 91% gap nt 29855-29871 | Istituto Zooprofilattico Sperimentale del Mezzogiorno ǀ TIGEM ǀ Grimaldi, Annunziata *et al* |
| GH | B.1.427**** | EPI_ISL_879977 | Tijuana (Mexico) | 2020-11 | 99% nt 29756 G->A | San Diego County Public Health Laboratory ǀ Andersen lab at Scripps Research ǀ SEARCH Alliance San Diego with Tracy Basler, Jovan Shephard, Brett Austin |
| GH | B.1.427**** | EPI_ISL_1016610 | California (USA) | 2021-02 | 97% gap nt 29865-29869 | Helix/Illumina ǀ Respiratory Viruses Branch, Centers for Disease Control and Prevention ǀ Cook, Howard *et al* |
| GH | B.1.429**** | EPI_ISL_753563 | California (USA) | 2020-12 | 100% | Clinical virology Laboratory, Children's Hospital Los Angeles ǀ Center for Personalized Medicine, Children's Hospital Los Angeles ǀ Gai et al |
| GH | B.1.429**** | EPI_ISL_955330 | Georgia (USA) | 2021-01 | 100% | GA Department of Public Health Laboratory ǀ Pathogen Discovery, Respiratory Viruses Branch, Division of Viral Diseases, Centers for Disease Control and Prevention ǀ Tao, Zhang *et al* |
| GH | B.1.429**** | EPI_ISL_956329 | Taoyuan (Taiwan) | 2021-01 | 100% | Laboratory Medicine ǀ Department of Laboratory Medicine, Lin-Kou Chang Gung Memorial Hospital ǀ Tsao, Gong *et al.* |

| | | | | | | |
|---|---|---|---|---|---|---|
| G | PL B.1.400 | EPI_ISL_753415 | Los Angeles (USA) | 2020-11 | 100% | Clinical virology Laboratory, Children's Hospital Los Angeles ǀ Center for Personalized Medicine, Children's Hospital Los Angeles ǀ Gai *et al* |
| G | PL B.1.237 | EPI_ISL_682338 | Free State (South Africa) | 2020-09 | 100% | NHLS Universitas Academic ǀ UFS Virology ǀ PA Bester, MM Nyaga *et al.* |
| G | PL B.1.5 | EPI_ISL_539541 | Catalunya (Spain) | 2020-06 | 100% | Hospital Clinic ǀ Instituto de Salud Carlos III ǀ M Iglesias-Caballero, M Molinero-Calamita *et al.* |
| G | PL B.1.221 | EPI_ISL_831985 | Stockholm (Sweden) | 2020-12 | 100% | ABC ǀ The Public Health Agency of Sweden ǀ Department of Microbiology, The Public Health Agency of Sweden |
| G | PL B.1.395 | EPI_ISL_859570 | Abu Dhabi (UAE) | 2020-08 | 100% | BTC, Khalifa University ǀ Al Safar *et al* |
| GR | PL B.1.1.28 | EPI_ISL_848623 | Para (Brazil) | 2020-04 | 100% | Evandro Chagas Institute ǀ MC Santos, AM Silva *et al.* |
| GR | PL B.1.1.7** | EPI_ISL_857469 | Singapore | 2021-01 | 99% nt 29790 A->G | National Public Health Laboratory, National Centre for Infectious Diseases ǀ TM Mak, S Octavia *et al* |
| GR | PL B.1.1.7** | EPI_ISL_763946 | England (UK) | 2020-12 | 100% | Oxford Viromics, NDM, Oxford University Hospitals ǀ COVID-19 Genomics UK (COG-UK) Consortium ǀ Golubchik T, Bonsall D *et al.* |
| GR | PL B.1.1.7** | EPI_ISL_679641 | England (UK) | 2020-11 | 99% nt 29850 A->G | Oxford Viromics, NDM, Oxford University Hospitals ǀ COVID-19 Genomics UK (COG-UK) Consortium ǀ Golubchik T, Bonsall D *et al.* |
| GR | PL B.1.1.7** | EPI_ISL_763844 | England (UK) | 2021-01 | 100% | Oxford Viromics, NDM, Oxford University Hospitals ǀ COVID-19 Genomics UK (COG-UK) Consortium ǀ Golubchik T, Bonsall D *et al.* |
| GR | PL B.1.1 | PI_ISL_412972 | Mexico (Mexico) | 2020-02 | 100% | Instituto Nacional de Enfermedades Respiratorias ǀ Instituto de Diagnostico y Referencia Epidemiologicos (INDRE) ǀ E Ramirez-Gonzalez, F Garces-Ayala *et al.* |

| | | | | | | |
|---|---|---|---|---|---|---|
| GR | PL B.1.1237 | EPI_ISL_479093 | England (UK) | 2020-05 | 100% | Oxford Viromics, NDM, University of Oxford; Oxford University Hospitals; Basingstoke and North Hampshire Hospital I COVID-19 Genomics UK (COG-UK) Consortium I T Golubchik, D Bonsall *et al.* |
| GR | PL B.1.1.229 | EPI_ISL_653791 | Bari (Italy) | 2020-11 | 100% | I.R.C.C.S. "S. De Bellis" - Ente Ospedaliero I Istituto Zooprofilattico Sperimentale della Puglia e della Basilicata I A Parisi, A Bianco *et al*. |
| GR | P.1.*** | EPI_ISL_906081 | Manaus (Brazil) | 2021-01 | 99% nt 29834 T->A nt 29858 T->A/T | Hospital Beneficiencia Portuguesa I Instituto Adolfo Lutz IC Tavares , C Regina *et al.* |
| GR | P.1.*** | EPI_ISL_875566 | Abruzzo (Italy) | 2021-01 | 99.5% nt 29834 T->A | SIESP L'AQUILA I Istituto Zooprofilattico Sperimentale dell'Abruzzo e Molise "G. Caporale" I A Lorusso, M Marcacci *et al.* |
| GV | PL B.1.177 | EPI_ISL_733500 | Ben Arous (Tunisia) | 2020-07 | 100% | Charles Nicolle Hospital, University of Tunis ElManar, National Observatory of New and Emerging Diseases I IBB Boubaker, S Trabelsi *et al*. |
| GV | PL B.1.189 | EPI_ISL_483514 | San Diego (USA) | 2020-06 | 100% | UC San Diego Center for Advanced Laboratory Medicine I Andersen lab at Scripps Research I SEARCH Alliance San Diego with David Pride, Ji H Shin |
| GV | P.1.177 | EPI_ISL_582841 | Orebro (Sweden) | 2020-08 | 100% | Orebro klinisk mikrobiologi I The Public Health Agency of Sweden I AM Linde, ML Karlberg *et al* |
| GV | PL B.1 | EPI_ISL_641555 | Toulouse (France) | 2020-05 | 100% | CHU Toulouse I CNR Virus des Infections Respiratoires I A Bal, G Destras *et al.* |
| GV | PL B.1.172 | EPI_ISL_842194 | England (UK) | 2020-10 | 100% | Oxford Viromics, NDM, University of Oxford; Oxford University Hospitals; Basingstoke and North Hampshire Hospital I COVID-19 Genomics UK (COG-UK) Consortium I T Golubchik, D Bonsall *et al.* |

| | | | | | | |
|---|---|---|---|---|---|---|
| L | PL B | EPI_ISL_434534 | Wuhan (China) | 2019-12 | 100% | National Institute for Viral Disease Control and Prevention, ǀ W Tan, R Lu *et al.* |
| L | PL B | EPI_ISL_812867 | Egypt | 2020-07 | 100% | Genomics Program, Children Cancer Hospital ǀ A Hatem, A Hadad *et al.* |
| L | PL B.6 | EPI_ISL_475025 | Jerusalem (Israel) | 2020-04 | 100% | Israel Central Virology laboratory ǀ N Zuckerman, ED Bucris *et al.* |
| L | PL B.3 | EPI_ISL_416488 | Zielonogorskie (Poland) | 2020-03 | 100% | ViroGenetics - BSL3 Laboratory of Virology; Human Genome Variation Research Group & Genomics Centre MCB; Bioinformatics Research Group Department of Virology ǀ A Milewska, E Pośpiech *et al.* |
| L | PL B | EPI_ISL_447608 | Frankfurt (Germany) | 2020-02 | 100% | Institute for Medical Virology, Goethe University Hospital Frankfurt ǀ T Toptan, S Hoehl *et al.* |
| O | PL B | EPI_ISL_406844 | Clayton (Australia) | 2020-01 | 97% gap nt 29749-29759 | Monash Medical Centre ǀ University of Melbourne, the Victorian Infectious Disease Reference Laboratory ǀ L Caly, T Seemann *et al.* |
| O | PL B | EPI_ISL_406031 | Kaohsiung (Taiwan) | 2020-01 | 100% | Centers for Disease Control, R.O.C. ǀ JR Yang, YC Lin *et al* |
| O | PL B | EPI_ISL_728153 | Aichi (Japan) | 2020-03 | 100% | Fujita Health University School of Medicine ǀ T Murata, Y Doi *et al.* |
| O | PL B.6.6 | EPI_ISL_508424 | Yavatmal (India) | 2020-05 | 99% nt 29868 G->A | Mahatma Gandhi Institute of Medical Sciences ǀ National Institute of Biomedical Genomics ǀ A Maitra, V Deotale *et al.* |
| O | PL B.1.1.1 | EPI_ISL_812837 | Egypt | 2020-07 | 100% | Genomics Program, Children Cancer Hospital ǀ A Hatem, A Hadad *et al.* |
| S | PL A.6 | EPI_ISL_447908 | Bangkok (Thailand) | 2020 | 100% | Siriraj hospital ǀ National Institute of Health ǀ O Pilailuk,H Navin *et al.* |
| S | PL A | EPI_ISL_812957 | South Korea | 2020-03 | 100% | Division of Pathogen Resource Management, Korea National Institute of Health, Korea Disease Control and Prevention Agency ǀ ST Kim, SY Kim *et* |

| | | | | | | |
|---|---|---|---|---|---|---|
| | | | | | | *al*. |
| S | PL A | EPI_ISL_779264 | Karachi (Pakistan) | 2020-06 | 99% nt 29742 G->A | Jamil-ur-Rahman Center for Genome Research, Dr. Panjwani Center for Molecular Medicine and Drug Research ǀ ; Shakeel, M Irfan *et al.* |
| S | PL A.2 | EPI_ISL_454503 | Nur-Sultan (Kazakhstan) | 2020-04 | 100% | RSE "National Center for Biotechnology" ǀ A Shevtsov, I Akhmetollayev *et al*. |
| S | PL A | EPI_ISL_859691 | Abu Dhabi (UAE) | 2020-09 | 99% nt 29742 G->A | BTC, Khalifa University ǀ Al Safar *et al.* |
| V | PL B.28 | EPI_ISL_428897 | St. Petersburg (Russia) | 2020-03 | 100% | State Research Center of Virology and Biotechnology VECTOR ǀ SA Bodnev, OV. Pyankov *et al*. |
| V | PL B | EPI_ISL_482335 | Oregon (USA) | 2020-04 | 100% | Providence St. Joseph Health Molecular Genomics Laboratory ǀ AK Dowdell, BD Piening *et al*. |
| V | PL B.40 | EPI_ISL_729947 | Lagos State (Nigeria) | 2020-05 | 100% | Nigeria Centre for Disease Control ǀ African Centre of Excellence for Genomics of Infectious Diseases, Redeemer's University ǀ Oluniyi P.E. *et* al |
| V | PL B | EPI_ISL_437303 | Styria (Austria) | 2020-04 | 100% | Diagnostic and Research Institute of Pathology, Medical University of Graz ǀ K Kashofer, P Regitnig *et al*. |
| V | PL B | EPI_ISL_457990 | Muscat (Oman) | 2020-03 | 100% | Oman-NIC ǀ S Al-Maruqi, F Zadjali *et al*. |
